# Physical education teacher education for intercultural citizenship in a traumatized society

**DOI:** 10.3389/fspor.2026.1936237

**Published:** 2026-10-07

**Authors:** Mahmood Sindiani, Devora Hellerstein

**Affiliations:** The Levinsky-Wingate Academic College, Wingate Institute, Netanya, Israel

**Keywords:** intercultural citizenship, intergroup contact, physical education teacher education (PETE), sport for development and peace (SDP), trauma-sensitive pedagogy

## Abstract

In divided and conflict-affected societies, higher education institutions offer opportunities for sustained intergroup encounter. Yet proximity alone does not produce meaningful interaction, trust, or cooperation. This challenge is particularly acute in Israel, where Arab and Jewish students enter higher education after years of segregated schooling, amid tensions and intensified fear, mistrust, and collective trauma following October 7, 2023, and the subsequent war. This perspective article argues that Physical Education Teacher Education (PETE) offers an underexplored space for fostering intercultural citizenship. It synthesizes relevant theoretical perspectives and evidence from studies by the authors and others to develop a framework for PETE in divided and traumatized societies. Integrating intergroup contact theory, intercultural citizenship, Sport for Development and Peace, and trauma-sensitive pedagogy, the framework positions PETE as a space for structured contact, embodied cooperation, civic engagement, and resilience. The framework is illustrated through the “Roaming Course,” an experiential, student-led PETE model combining on-campus learning, encounters in diverse communities, shared physical activity, and guided reflection. Drawing on studies involving Arab and Jewish PETE students in Israel, the article shows how structured, discipline-based contact may narrow the gap between willingness to engage and actual intergroup interaction while strengthening students’ capacity to cope with social tension and collective trauma. It demonstrates the value of embedding intercultural citizenship within core curricula rather than relying on extracurricular interventions. It concludes that PETE can prepare future educators to act as agents of social connection, inclusion, and resilience in divided societies.

## 1 Introduction

Intergroup relations in deeply divided societies are shaped by long histories of segregation, conflict, and structural inequality, which sustain prejudice and limit opportunities for meaningful contact between groups (1, 2). Education systems in such contexts often reproduce rather than challenge these divisions, as they are embedded within broader sociopolitical structures that perpetuate inequality and social stratification (3–5).

These insights have been offered mainly as a result of research on schooling systems that socialize children into these structures. However, Higher Education Institutions (HEIs) occupy a paradoxical position within these societies. While they constitute one of the few shared social spaces available to young adults, with the potential to enable meaningful intergroup encounters, they often reproduce existing divisions through patterns of self-segregation and limited intercultural interaction among students who have already been socialized into divisions (6–9). Research consistently documents a gap between students' expressed openness to diversity and their actual intergroup behavior, a phenomenon that becomes especially salient in conflict-affected settings (10–13). This discrepancy may be shaped by perceived intergroup threat, intergroup anxiety, entrenched group boundaries, and limited access to structured environments that support meaningful and sustained interaction across communities (14–17). Even when students endorse intercultural engagement in principle, negative expectations about intergroup encounters, misperceptions of ingroup and outgroup contact norms, and identity-related concerns, such as fear of rejection or concern about how one's group will be perceived, may inhibit the initiation and development of actual intergroup contact (18–21).

In Israel, this paradox is intensified by a structurally segregated schooling system in which the majority of Jewish and Arab students are educated apart from one another throughout their K-12 education (22, 23). For many students, entry into HEIs in Israel marks the first sustained encounter with members of the other group. Yet, without intentional pedagogical design, these encounters often remain superficial or are actively avoided (10, 24, 25).

While the attempt to form intergroup contact spaces and fulfil the potential of higher education contexts has always been challenging, prior to October 2023, several indicators suggested increasing opportunities for Arab–Jewish interaction and participation across educational, professional, and civic domains, accompanied by reports of improving attitudes in some higher education contexts (10). This trajectory was abruptly disrupted on October 7, 2023, when the terrorist group Hamas claimed the lives of 1,200 Israeli citizens and foreign nationals and abducted 241 people including infants and the elderly. These events and the subsequent war destabilized the fragile contact spaces that had been formed. Collective trauma, fear, loss, and heightened intergroup threat reshaped campus climates, deepening polarization and undermining trust (26, 27). In this context, the question is no longer whether intergroup contact is desirable, but under what conditions it remains possible and constructive.

This article argues that Physical Education Teacher Education (PETE) offers a uniquely powerful yet underexplored pedagogical space for addressing these challenges. Building on contact theory, intercultural citizenship, and sport for development and peace (SDP) scholarship, and grounded in empirical work conducted by the authors and colleagues, the article proposes a trauma-sensitive, contact-based pedagogical framework for PETE in divided and traumatized societies.

### 1.1 The role of higher education institutions as contact zones

Higher education institutions (HEIs) may be understood as potential social arenas that exist outside students’ segregated home communities and prior schooling trajectories, where new forms of interaction, identity negotiation, and civic engagement become possible. Conceptually, HEIs can be framed as providing the potential to create a *third space.* Based on Bhabha's (28) conceptualization of the third space, such environments can be understood as sites where individuals from different cultural groups meet, negotiate meanings, and develop a hybrid culture characterized by new ways of understanding themselves and others. Rather than treating cultures as fixed or separate, the third space emphasizes the transformations that emerge through interaction, dialogue, and shared experience. Expanding this view, Soja's (29) concept of *Thirdspace* posits that space is not merely physical, but is actively shaped by lived experiences, relationships, and social meanings. In educational research, Gutiérrez (30) applies this framework to describe learning spaces where diverse languages, identities, and ways of thinking converge. These spaces ultimately support dialogue, reflection, and change by helping participants move beyond rigid group labels to build new, shared understandings.

HEIs have the potential to provide settings that foster the formation of third spaces by suspending, at least partially, rigid social divisions and enabling dialogic relational encounters across difference. These environments can advance processes of reflexivity, boundary-crossing, and hybrid identity formation, allowing students to construct new forms of belonging that are not wholly determined by pre-existing group affiliations. However, the realization of this potential is neither automatic nor guaranteed. Without intentional pedagogical design and structured opportunities for sustained interaction, these spaces often reproduce patterns of parallel coexistence rather than meaningful engagement, thereby limiting the transformative potential of intercultural encounters. Evidence from both Israeli and international higher education settings suggests that diversity alone does not guarantee meaningful intercultural engagement, underscoring the importance of intentionally designed educational frameworks that support sustained interaction across group boundaries (7, 10, 22, 31).

### 1.2 Aims of the article

Building on the theoretical lenses of intergroup contact, intercultural citizenship, and Sport for Development and Peace (SDP), this article pursues two interrelated aims. First, it seeks to conceptualize the contribution of Physical Education Teacher Education (PETE) to intercultural engagement in a deeply divided and traumatized society. Second, it proposes and examines a pedagogical model that supports not only structured intergroup contact, but also students' capacity to cope with collective trauma through embodied, collaborative, and reflective learning processes. In doing so, the article positions PETE as a significant educational space where social engagement, psychosocial wellbeing, and emotional resilience are mutually reinforced.

## 2 Theoretical framework

### 2.1 Intergroup contact in divided and traumatized contexts

The contact hypothesis (32) remains one of the most influential frameworks for understanding prejudice reduction and intergroup relations. Allport argued that contact can improve relations when it occurs under conditions of equal status, common goals, cooperation, and institutional support. Subsequent meta-analyses have largely confirmed the positive effects of intergroup contact on reducing prejudice and improving intergroup attitudes (14, 33). At the same time, scholars have highlighted important limitations, noting that the effects of contact may be constrained by methodological issues (34) and by contexts characterized by power asymmetries, structural inequalities, and ongoing conflict (35). Nevertheless, even these studies generally report positive outcomes associated with intergroup contact, although the magnitude and nature of these effects may vary across contexts.

Recent research emphasizes that in conflict-affected societies, intergroup contact cannot be understood as a discrete interpersonal encounter alone, but rather as a process deeply embedded within broader sociopolitical structures and collective emotional climates (1, 2, 35, 36). In such contexts, contact is shaped by entrenched power relations, competing national narratives, and institutionalized inequalities, all of which influence how interactions are perceived and experienced. Moreover, shared histories of violence and collective trauma infuse intergroup encounters with heightened emotions, often activating fear, mistrust, and defensive identity mechanisms. These dynamics are closely linked to what (36) conceptualizes as the *ethos of conflict*, a system of shared societal beliefs that justifies in-group goals, delegitimizes the out-group, and reinforces a persistent sense of threat. Within such a framework, intergroup contact is not approached as a neutral or purely cooperative interaction but is filtered through deeply internalized narratives and collective memories. Accordingly, even under formally optimal conditions, contact may reproduce suspicion, avoidance, or symbolic distancing rather than genuine engagement.

Collective trauma adds a further layer to this sociopolitical context. It refers not only to the consequences of a catastrophic event for those directly exposed to it but also to the ways in which the event becomes embedded in a group's collective memory, identity, and systems of meaning (37). Such collective processes may sustain perceptions of vulnerability and threat across time. Their effects are not uniform, however, and collective trauma should not be equated with a clinical trauma response among all members of an affected group.

At the individual level, trauma exposure, particularly when accompanied by persistent posttraumatic stress symptoms, may heighten physiological arousal and vigilance, orient attention and interpretation toward potential threat, and reduce flexibility in cognitive and social-cognitive processing under stress (38–40). During intergroup encounters, these responses may increase sensitivity to ambiguous social cues, make perspective-taking more difficult, and encourage withdrawal or avoidance (41). Trauma may therefore influence intergroup contact through the interaction between collective threat-based narratives and individual cognitive, emotional, and physiological responses. Importantly, these processes should be understood as possible responses rather than as uniform consequences of trauma exposure.

These interacting societal and individual processes help explain why armed conflict and collective trauma may heighten perceived threat, weaken trust, and widen the gap between willingness for contact and actual intergroup engagement (2, 42). Recent research in Israeli HEIs demonstrates that expressed willingness for intergroup closeness does not necessarily translate into sustained interaction and that periods of violent escalation may further affect both willingness and engagement (10, 27). These findings reinforce the need to consider not only whether contact occurs but also how intergroup encounters are structured and how participants' emotional, cognitive, and physiological responses are recognized and supported.

### 2.2 Intercultural citizenship

Intercultural communicative competence (ICC) is commonly defined as the ability to interact effectively and appropriately across cultural differences, encompassing a combination of knowledge, skills, attitudes, and reflexivity that enable individuals to communicate and collaborate in diverse settings (43). Central to this framework is the development of openness, curiosity, and the capacity to decenter one's own perspective in order to understand and engage with others. Building on ICC, intercultural citizenship emphasizes individuals' capacity not only to understand and interact effectively with people from different cultural backgrounds but also to engage critically with social issues and participate collaboratively in civic action across cultural boundaries (44–46). It combines intercultural understanding with democratic participation, encouraging learners to reflect on multiple perspectives, challenge assumptions, and work with others toward shared social goals. It thus shifts the focus from individual *competence* to *commitment* for democratic participation and social transformation.

In divided societies, intercultural citizenship requires educational spaces that enable students to move beyond passive coexistence toward meaningful engagement and shared accountability. This involves not only facilitating intergroup contact, but also creating conditions for critical dialogue, perspective-taking, and collaborative action across group boundaries. HEIs are increasingly conceptualized as potential sites for fostering such development, given their role as pluralistic environments where diverse identities intersect. However, studies consistently demonstrate that mere exposure to diversity is insufficient, and that structured and unstructured institutional initiatives promoting intergroup collaborative work are necessary (47–50).

### 2.3 Sport, physical education, and peacebuilding

Sport fosters intergroup contact by leading participants to collaborate around shared goals following equal rules, as advocated by the contact hypothesis. Recognizing this potential, the UN's 2030 Agenda identified sport as a tool for development and peace. The SDP movement seeks to leverage sport to enhance social and civic conditions, including education, social inclusion, and conflict resolution particularly in contexts of division (51–54). As such, sport can serve as an important tool for the development of intercultural citizenship. Within this framework, approaches such as Olympic education and fair play pedagogy further emphasize values of respect, mutual recognition, empathy, and ethical responsibility. By promoting principles such as fairness, rule adherence, and respect for opponents, sport-based educational models can create structured environments in which intergroup interaction is guided by commonly accepted standards, thereby reducing uncertainty and facilitating trust-building.

Embodied cooperation, which is integral to sport, has been shown to reduce intergroup anxiety and promote prosocial behavior, often through non-verbal and relational pathways (55). These processes operate through several interrelated mechanisms. First, coordinated physical activity (e.g., team play, synchronized movement, joint goal pursuit) fosters *behavioral synchrony*, which has been linked to increased feelings of affiliation, trust, and social bonding. Second, shared physical goals shift attention away from group identities toward task-oriented collaboration, thereby reducing the salience of intergroup boundaries. Third, the non-verbal nature of many sport interactions enables communication that bypasses linguistic and cultural barriers, allowing participants to develop implicit forms of understanding and mutual responsiveness. Finally, repeated positive embodied interactions may gradually reshape emotional associations with out-group members, reducing anxiety and fostering more positive expectations of future contact.

When integrated into educational contexts, sport-based pedagogies can therefore support both social learning and emotional regulation, a particularly critical consideration in traumatized and conflict-affected societies.

Sport for Development and Peace contributes an embodied and relational medium through which common goals, cooperation, and interpersonal connection may be cultivated. Trauma-sensitive pedagogy, in turn, draws attention to participants' emotional, cognitive, and physiological responses and to the need for safety, choice, careful facilitation, and guided reflection. Integrated within PETE, these perspectives suggest that structured contact, embodied cooperation, civic learning, and resilience are not separate outcomes but mutually reinforcing processes that must be intentionally supported.

Taken together, intergroup contact theory, intercultural citizenship, SDP, and trauma-sensitive pedagogy provide complementary foundations for educational engagement in divided and conflict-affected societies. Intergroup contact theory identifies the conditions that can support constructive interaction across group boundaries, while intercultural citizenship gives this process a broader civic purpose by connecting it to critical reflection, agency, social responsibility, and collaborative action. SDP contributes an embodied and relational medium through which common goals, cooperation, and interpersonal connection may be cultivated. Trauma-sensitive pedagogy, in turn, draws attention to participants' emotional, cognitive, and physiological responses and to the need for safety, choice, careful facilitation, and guided reflection. Integrated within PETE, these perspectives suggest that structured contact, embodied cooperation, civic learning, and resilience are not separate outcomes but mutually reinforcing processes that must be intentionally supported.

### 2.4 Well-being, social connectedness, and resilience

While intergroup contact and intercultural citizenship provide important foundations for understanding relationship-building across social divides, increasing attention has been directed toward the role of sport and education in promoting psychosocial wellbeing, particularly in contexts affected by conflict and collective trauma. Wellbeing is commonly understood as a multidimensional construct encompassing psychological, emotional, and social functioning, including feelings of belonging, connectedness, purpose, competence, and resilience (56, 57).

Research increasingly recognizes that sport-based educational environments can contribute to psychosocial wellbeing by fostering social inclusion, supportive relationships, empowerment, and community engagement (51, 53, 58). These outcomes are especially relevant in divided and conflict-affected societies, where collective trauma can undermine trust, belonging, and social cohesion while increasing perceptions of uncertainty and intergroup threat (37, 42).

A growing body of literature further suggests that social connectedness and a sense of belonging are among the strongest predictors of wellbeing and resilience among young adults. Individuals who experience meaningful relationships and perceive themselves as valued members of their social environments report better psychological adjustment, greater coping capacity, and stronger overall wellbeing (59, 60).

Within PETE, shared physical activity, cooperative learning experiences, peer support, and guided reflection may serve as mechanisms through which wellbeing is strengthened. Repeated opportunities for collaboration and mutual reliance can foster social connectedness, emotional safety, and confidence in engaging across cultural differences. In this sense, intercultural citizenship, wellbeing, and resilience should not be viewed as separate educational outcomes but rather as interconnected dimensions of a broader developmental process through which students learn to engage constructively with diversity while strengthening their capacity to cope with social adversity.

## 3 The unique role of physical education teacher education

PETE occupies a distinctive pedagogical position within higher education. Its emphasis on embodied, experiential, and collaborative learning differentiates it from predominantly verbal, text-based and mainly theoretical disciplines. PETE involves cooperative physical activities, shared challenges, teamwork, and experiential practical learning, which can provide opportunities to create conditions consistent with Allport's (32) conditions for effective intergroup contact. However, it is important to emphasize that such outcomes do not necessarily emerge automatically. They depend on the intentional structuring of activities around common goals, cooperation, inclusion, and guided reflection that enables participants to examine and make sense of their interactions. Without deliberate pedagogical guidance and institutional support, physical activity settings may instead reinforce competition and exclusion or heighten existing group boundaries. Emerging evidence suggests that, when carefully designed and facilitated, shared physical activity in PETE may provide a mode of interaction that is less dependent on verbal fluency, thereby supporting engagement across linguistic and cultural differences when direct dialogue feels difficult or threatening (25). Similar findings from SDP and multicultural PETE research indicate that physical activity contexts provide powerful opportunities for meaningful intercultural engagement and the development of intercultural competence (61–63). However, these benefits depend on pedagogical approaches that emphasize cooperation, inclusion, and critical reflection rather than competition alone.

Within the Israeli context, these dynamics take on particular significance. Empirical studies in PETE programs show that PETE interventions that structure collaboration between Jewish and Arab students can foster empathy, trust, and critical awareness, even during periods of heightened sociopolitical tension (64). These findings suggest that physically grounded, cooperative learning environments may serve as especially robust platforms for intergroup engagement in settings marked by conflict and division.

Moreover, PETE students are future educators whose professional identities extend beyond technical instruction. As teachers, coaches, and community leaders, they are positioned to influence school cultures and intergroup relations among their future students.

Based on the above-established assumptions, we argue that PETE can serve as a meaningful mediating space when structured intergroup contact is supported by trauma-sensitive pedagogy and intercultural citizenship education. Rather than treating intergroup contact and coping with trauma as separate objectives, the framework recognizes their interdependence. Collective trauma limits students' emotional availability for contact, while meaningful engagement can, under supportive conditions, contribute to intercultural competence and resilience.

Longitudinal evidence from Israeli PETE programs illustrates this dynamic. In the context of the October 7, 2023 Hamas attacks on Israeli communities and the ensuing prolonged and highly violent escalation in the Israeli-Palestinian conflict, which were accompanied by widespread societal trauma, heightened insecurity, and increased intergroup tensions within Israel, patterns of intergroup interaction shifted markedly. While this period was associated with a decline in willingness and actual intergroup engagement among Jewish students, Arab students demonstrated relatively higher levels of openness and interaction, pointing to asymmetric responses to trauma and perceived threat (27, 65, 66).‏ Such divergence aligns with broader patterns observed in conflict-affected societies, where heightened threat perceptions may lead dominant-group members to withdraw from intergroup contact, whereas minority-group members may maintain or even increase engagement, reflecting differing social positions, vulnerabilities, and orientations toward shared civic life (67–69).

These findings underscore the need for pedagogical designs that maintain Allport's principle of equal status while simultaneously recognizing that students enter intergroup encounters with different experiences of vulnerability, loss, and perceived threat. In conflict-affected societies, effective educational practice requires balancing equality of participation with sensitivity to diverse lived experiences. This challenge provides an important rationale for the trauma-sensitive pedagogical model presented in the following section.

## 4 The pedagogical model: the “roaming course”

The Roaming Course represents a practical application of the framework proposed in this article. Within PETE, it functions not only as a setting for intergroup contact, but as a structured pedagogical model designed to cultivate intercultural citizenship, trauma-sensitive engagement, and students' professional identities as socially responsible educators and agents of change.

### 4.1 The model's conceptual underpinnings

The model integrates three complementary theoretical dimensions: structured intergroup contact, intercultural citizenship, and sport-based embodied pedagogy. These dimensions are integrated through trauma-sensitive practice and an explicit emphasis on agency, which function as mediating pedagogical principles.

Structured intergroup contact provides the foundation of the model. The course creates sustained opportunities for Arab and Jewish students to work cooperatively under conditions of equal status, shared goals, institutional support, and interdependence. Rather than assuming that contact naturally emerges within higher education, interaction is intentionally designed through mixed-group collaboration and shared educational responsibilities.

Intercultural citizenship extends contact beyond prejudice reduction toward civic engagement and social responsibility. Students are encouraged not only to understand different perspectives but also to critically examine social divisions, reflect on their own identities and assumptions, and consider their responsibilities as future educators. Intercultural learning is therefore linked to action, positioning students as contributors to more inclusive educational and social environments.

Sport-based embodied pedagogy provides the primary pedagogical medium through which these processes unfold. Shared physical activity enables students to cooperate before engaging in potentially difficult dialogue, creating opportunities for trust-building through movement, teamwork, and shared achievement. As students jointly design and facilitate sport activities for children from diverse communities, they experience how physical education can become a practical vehicle for inclusion and intercultural engagement.

These three dimensions are supported by trauma-sensitive pedagogy, which recognizes that intergroup encounters in conflict-affected societies are emotionally and politically situated. Rather than treating contact as inherently positive, the model prioritizes psychological safety, gradual engagement, structured reflection, and acknowledgement of participants' lived experiences. This supportive environment enables students to engage constructively with difference despite uncertainty, fear, or discomfort.

Closely connected to trauma-sensitive practice is the development of agency. Drawing on Biesta et al. (70) and Priestley et al. (71), the model positions students as active participants who exercise professional judgment, take initiative, and assume responsibility for shaping relationships within and beyond the course. Agency is therefore understood not as an individual trait but as a capacity cultivated through participation, reflection, and meaningful action within supportive educational structures.

### 4.2 The model in practice

The Roaming Course operationalizes these principles through the integration of theoretical learning, experiential engagement, collaborative practice, and structured reflection. The program combines campus-based sessions with field experiences in culturally diverse communities chosen by the students. On-campus sessions introduce the theoretical foundations of intergroup contact, intercultural citizenship, and SDP while preparing students for field engagement. Mixed-student groups then apply the theories by organizing visits to diverse educational and community settings of their choice where students interact with local stakeholders, and examine real-world examples of social division and cooperation. Students then move from learners to practitioners by collaboratively designing, implementing, and evaluating sport-based educational activities for children from diverse backgrounds. Through these experiences they learn not only about intercultural relations but also how to facilitate them professionally using physical education.

Throughout the process, reflective journals and facilitated discussions connect experience with theory. Reflection enables students to process emotional responses, critically examine assumptions, make sense of challenging encounters, and consider the implications of these experiences for their future professional practice.

The model follows a developmental progression. Initial embodied cooperation reduces anxiety and establishes interpersonal trust. As trust develops, students become increasingly willing to engage in dialogue, perspective-taking, and critical reflection. Through repeated cycles of experience and reflection, they gradually assume greater responsibility for planning, facilitating, and evaluating intercultural learning experiences. Figure 1 presents the integrated pedagogical model underpinning the Roaming Course and illustrates the relationships among structured intergroup contact, intercultural citizenship, embodied pedagogy, trauma-sensitive practice, agency, and developmental outcomes.

**Figure 1:**
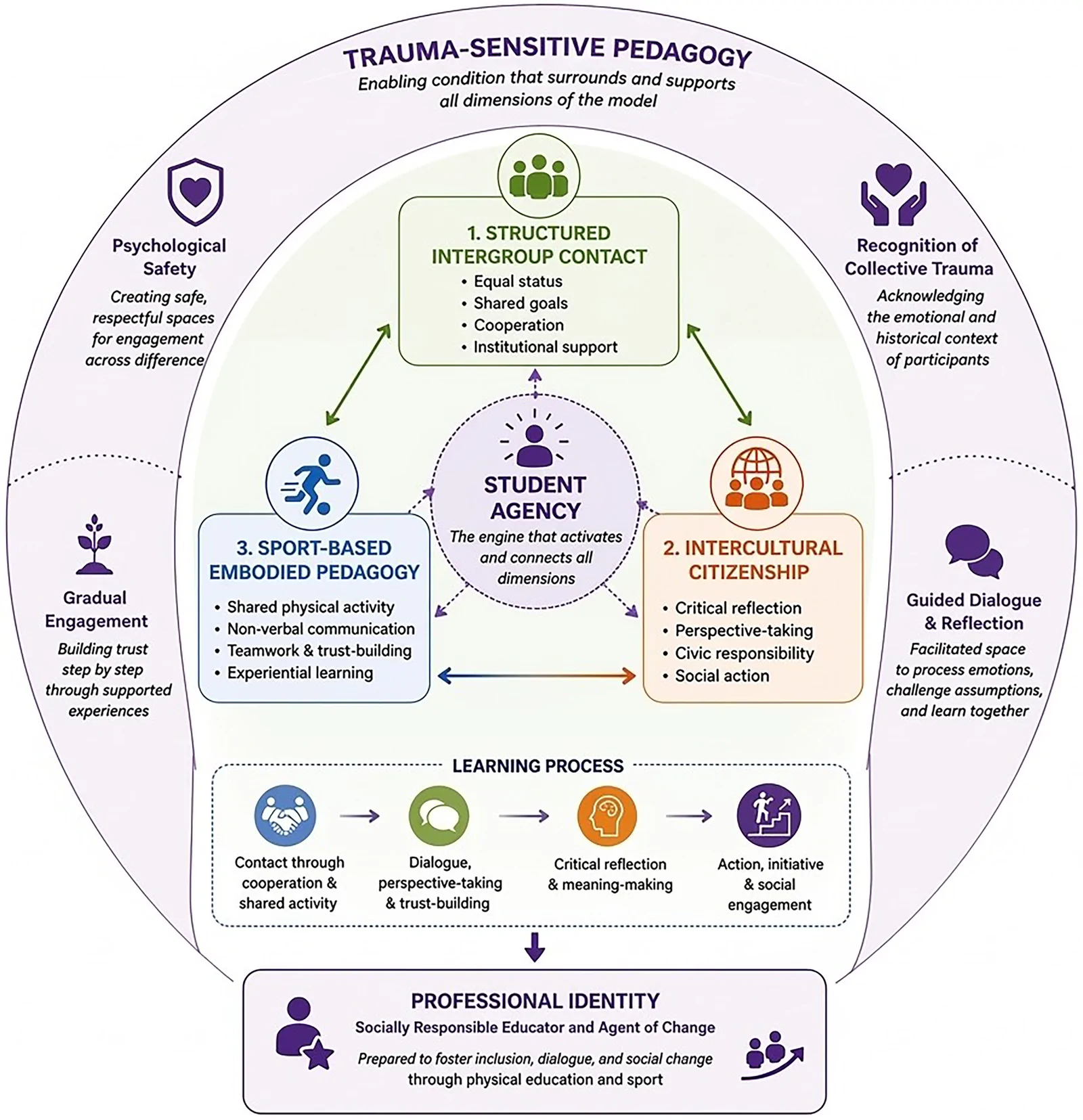
The roaming course pedagogical model for intercultural citizenship in PETE.

### 4.3 Developmental outcomes

The model fosters development at three interconnected levels. At the interpersonal level, students develop more meaningful intergroup relationships characterized by reduced prejudice, greater trust, empathy, and openness toward members of other communities. While it is not part of the requirements, students often choose to visit communities that represent their identities as an opportunity to teach their peers about and experience their own cultures. This process enables students to better understand and express their own identities and may strengthen their sense of belonging within the higher education setting and, more broadly, within society.

At the intercultural level, students strengthen intercultural citizenship through increased perspective-taking, critical cultural awareness, and dialogue skills, both within and beyond the higher education setting. Physical engagement with diverse “real-life” communities and settings exposes students to social and cultural realities that many would otherwise be unlikely to encounter.

At the professional level, students develop a broader conception of physical education teaching. Rather than viewing themselves primarily as instructors of physical skills, they increasingly understand their professional role as facilitating inclusion, dialogue, and social responsibility through sport. This evolving sense of professional agency enables them to recognize physical education as a means of creating more equitable and cohesive educational environments.

Psychosocial wellbeing is integral to this developmental process. The social connectedness, sense of belonging, emotional safety, and supportive peer relationships fostered through sustained cooperative engagement can create conditions that enable students to participate meaningfully in intergroup encounters, engage in dialogue across difference, and develop greater confidence in their capacity to act within diverse educational and social environments. At the same time, these experiences may themselves strengthen resilience and positive psychological and social functioning, particularly in conflict-affected societies where stress and perceived intergroup threat can inhibit sustained engagement across social divides.

Evidence that belonging, supportive interpersonal relationships, and community connectedness are closely associated with resilience and wellbeing among young adults facing adversity further supports this relationship (59, 60). Wellbeing can therefore be understood as both an enabling condition for, and a potential consequence of, the interpersonal, intercultural, and professional development promoted through the Roaming Course.

Ultimately, the Roaming Course positions PETE as more than professional preparation for teaching physical education. It demonstrates how teacher education can become a space in which future educators develop the knowledge, skills, and agency required to foster intercultural citizenship and social cohesion in divided societies, processes that may in turn contribute to greater psychosocial wellbeing.

## 5 Empirical evidence supporting the model

Qualitative findings from the Roaming Course indicate shifts in attitudes, perceptions, and relationships among participants. Students report increased empathy, trust, and willingness to engage with peers from other groups, often attributing these changes to shared physical experiences that felt less threatening than direct political discussion (64). This shared embodied experience established trust which laid the foundations for dialogue in a safe environment.

Quantitative data further support the model. Longitudinal and repeated cross-sectional studies demonstrate a persistent gap between willingness for contact and actual interaction in HEIs, a gap that narrows significantly among students who participate in structured, collaborative PETE activities (10, 27).

These findings align with broader literature emphasizing the importance of sustained, high-quality contact over mere exposure (72, 73). They further support the current understanding that sport does not automatically produce conflict resolution or reconciliation. Rather, the evidence suggests that sport is most effective when embedded within structured, reflective, and educationally guided processes (52, 58). The Roaming Course pedagogical model similarly offers an integrative, structured program grounded in reflection, agency, and guided intergroup engagement.

However, much of the existing literature examines short-term interventions, often designed for children and youth. The Roaming Course extends this work by situating sport-based intergroup contact within a four-year PETE degree program for young adults preparing to become educators. In this model, the PETE student is positioned at the center of the process: first as a participant in structured intergroup contact, then as an emerging educator capable of shaping similar encounters for future pupils. In this sense, the model builds on Sugden and Tomlinson's (52) “ripple effect,” suggesting that the transformation of PETE students may later influence the children they teach, the school communities in which they work, and, ultimately, broader processes of social cohesion.

In this sense, the proposed framework also aligns with existing research on HEIs as potential spaces for establishing contact zones, while offering a PETE-specific contribution. Unlike dialogue-only interventions offered at HEIs, PETE integrates embodied cooperation, emotional regulation, and professional identity formation within an integrated pedagogical model. This integration is particularly valuable in traumatized societies, where verbal engagement alone may be insufficient or even counterproductive due to heightened emotional sensitivity, mistrust, and fear.

Initiatives such as the aChord Center for Social Psychology for Social Change provide an important model for structured intergroup contact in Israel. Drawing on social-psychological principles, aChord develops and implements interventions aimed at reducing prejudice, fostering shared society, and addressing barriers to intergroup trust through dialogue-based and attitudinal change programs. These initiatives have demonstrated significant benefits, particularly in increasing awareness, improving intergroup attitudes, and creating structured opportunities for meaningful encounters across group boundaries (65). However, much like sports interventions, such programs are often implemented as discrete or supplementary interventions, and their impact may be limited in terms of sustaining long-term behavioral change, embedding everyday interaction practices, and shaping participants' professional identities.

In contrast, PETE embeds intergroup contact within core disciplinary curricula and professional training, thereby extending its influence beyond episodic encounters. Through continuous, practice-based engagement, students not only participate in intergroup interaction but also develop the pedagogical skills and a conscious sense of responsibility required to enact these practices in their future roles as educators. In this sense, PETE addresses key limitations of stand-alone interventions by fostering continuity, repetition, and integration between embodied experience, critical reflection, and identity formation over time.

This pedagogical integration supports the transition from passive coexistence to active intercultural citizenship by moving beyond mere exposure to diversity toward sustained, practice-oriented engagement. Rather than viewing diversity as a context to be tolerated, students are positioned as active agents capable of shaping inclusive environments, from the basketball court or football field, rippling to broader circles of the community, with the aim of building a inclusive and cohesive society. Through repeated experiences of cooperation, structured reflection, and engagement with real-world communities, they develop the capacity to initiate dialogue, navigate conflict constructively, and promote integrative practices in educational and civic settings.

Viewed through a wellbeing perspective, these findings suggest that the benefits of the Roaming Course extend beyond improved intergroup attitudes. Students' accounts frequently describe increased feelings of connection, trust, mutual support, and belonging. These experiences are widely recognized as important contributors to psychosocial wellbeing and resilience. Although wellbeing was not directly measured in the studies reviewed, the relational outcomes observed are consistent with theoretical models emphasizing the protective role of belonging and social connectedness in promoting wellbeing and positive adaptation (59, 60).

Future research should incorporate direct measures of wellbeing, belonging, resilience, and social support in order to more systematically examine the contribution of PETE-based interventions to psychosocial development in divided and traumatized societies.

## 6 Implications

For teacher education, the findings underscore the potential of discipline-specific experiential pedagogies to prepare educators as agents of social change while simultaneously promoting psychosocial wellbeing. PETE programs can intentionally cultivate competencies related to inclusion, intergroup dialogue, belonging, resilience, and social-emotional development alongside professional teaching skills.

The framework further suggests that structured and sustained sport-based educational experiences may contribute to broader goals of social inclusion, community wellbeing, and social cohesion. Through embodied cooperation, reflective practice, and meaningful engagement across cultural differences, students develop interpersonal and civic capacities that may extend beyond the higher education setting into schools and communities.

At the policy and institutional level, the article reinforces the responsibility of HEIs in divided societies to embed structured, trauma-sensitive, and wellbeing-oriented contact opportunities within academic curricula. Such initiatives may contribute not only to improved intergroup relations but also to the psychological and social wellbeing of students navigating conflict-related stress and uncertainty.

## 7 Conclusion

In societies marked by deep division and collective trauma, education cannot remain neutral or sustain divisions. This article has argued that PETE offers a powerful yet underutilized space for fostering intercultural citizenship through structured intergroup contact and trauma-sensitive pedagogy. Drawing on theory, empirical evidence, and the authors' own work, it demonstrates how embodied cooperation, when intentionally designed and institutionally supported, can sustain engagement even under conditions of profound sociopolitical strain.

Reimagining PETE in this way positions PE teachers not only as instructors of movement, but as facilitators of connection, understanding, and shared futures in divided societies. Perhaps the simplest image of the Roaming Course is that of students standing in a circle, holding hands before beginning a cooperative game. At first glance, they appear to be preparing for a physical activity. Yet something more profound is taking place. The circle creates connection before conversation, trust before dialogue, and cooperation before consensus. By holding hands, students are not only learning about movement, posture, or teamwork; they are experiencing what it means to rely on one another across difference. In that moment, physical education becomes more than the teaching of movement. It becomes a pedagogy of human connection, wellbeing, resilience, and social responsibility. In divided and traumatized societies, such experiences may serve not only as foundations for intercultural engagement but also as resources for individual and collective wellbeing. Sometimes, changing hearts and minds begins not with words, but with the simple act of reaching out and taking another person's hand.

## Data Availability

The original contributions presented in the study are included in the article/Supplementary Material, further inquiries can be directed to the corresponding author.
